# The Stimulation of CD147 Induces MMP-9 Expression through ERK and NF-κB in Macrophages: Implication for Atherosclerosis

**DOI:** 10.4110/in.2009.9.3.90

**Published:** 2009-06-30

**Authors:** Ju-Young Kim, Won-Jung Kim, Ho Kim, Kyoungho Suk, Won-Ha Lee

**Affiliations:** 1 The School of Life Sciences and Biotechnology, Kyungpook National University, Daegu 702-701, Korea.; 2 Department of Pharmacology, School of Medicine, Kyungpook National University, Daegu 702-701, Korea.

**Keywords:** macrophage, atherosclerosis, inflammation, CD147, cyclophilin A

## Abstract

**Background:**

CD147, as a cellular receptor for cyclophilin A (CypA), is a multifunctional protein involved in tumor invasion, inflammation, tissue remodeling, neural function, and reproduction. Recent observations showing the expression of CD147 in leukocytes indicate that this molecule may have roles in inflammation.

**Methods:**

In order to investigate the role of CD147 and its ligand in the pathogenesis of atherosclerosis, human atherosclerotic plaques were analyzed for the expression pattern of CD147 and CypA. The cellular responses and signaling molecules activated by the stimulation of CD147 were then investigated in the human macrophage cell line, THP-1, which expresses high basal level of CD147 on the cell surface.

**Results:**

Staining of both CD147 and CypA was detected in endothelial cell layers facing the lumen and macrophage-rich areas. Stimulation of CD147 with its specific monoclonal antibody induced the expression of matrix metalloproteinase (MMP)-9 in THP-1 cells and it was suppressed by inhibitors of both ERK and NF-κB. Accordingly, the stimulation of CD147 was observed to induce phosphorylation of ERK, phosphorylation-associated degradation of IκB, and nuclear translocation of NF-κB p65 and p50 subunits.

**Conclusion:**

These results suggest that CD147 mediates the inflammatory activation of macrophages that leads to the induction of MMP-9 expression, which could play a role in the pathogenesis of inflammatory diseases such as atherosclerosis.

## INTRODUCTION

CD147 (EMMPRIN/basigin/HAb18G/neurothelin/M6/TCSF), which has two immunoglobulin-like extracellular domains, is a multifunctional transmembrane glycoprotein with short (39 amino acids long) intracellular domain (1). CD147 plays a critical role in many pathological and physiological processes involving a variety of cell types such as various cancer cells, leukocytes, fibroblasts, and endothelial cells (2-7). As a tumor-derived MMP inducer, CD147 stimulates fibroblast and endothelial cells to facilitate tumor invasion, metastasis, and angiogenesis (7). In addition, CD147 enhances angiogenesis through stimulation of the production of vascular endothelial growth factor (VEGF) (8). The expression of CD147 has been shown to be induced in activated leukocytes such as granulocytes, lymphocytes, and macrophages (4). Stimulation of CD147 in leukocytes is believed to be involved in inflammatory processes associated with lung injury, rheumatoid arthritis (RA), chronic liver disease, heart failure, and atherosclerosis (9-13).

The ligands for CD147 were identified to be the two cyclosporin A binding proteins: cyclophilin A and B (CypA and CypB) (14,15). A secreted form of CypA, which are expressed by smooth muscle cells (SMCs) and macrophages during inflammatory conditions (16-18), has been shown to have cytokine-like functions (17,19). The expression of CypA and CD147 was detected in synovial macrophages of RA patients and stimulation of CD147 induced NF-κB-mediated expression of MMP-9 and proinflammatory cytokines and enhanced cell migration in macrophages (20,21). Accordingly, blocking the interaction between CD147 and CypA in a collagen-induced arthritis model resulted in a significant reduction in arthritic symptoms (22). Furthermore, CypA has been shown to have chemoattractant activity toward CD4^+^ T cells, which up-regulate the expression of CD147 after activation (23).

Although CD147 has been shown to be expressed by macrophages in atherosclerotic plaques (11) and in patients with acute myocardial infarction (24), the expression pattern and role of CD147 in relation to CypA has not been investigated simultaneously in the context of atherosclerosis. In this manuscript, the expression patterns of CD147 and CypA were compared in human atherosclerotic plaques and the role of CD147, in relation to CypA, was investigated in macrophage activation and cell signaling.

## MATERIALS AND METHODS

### Monoclonal antibodies, cell lines, and reagents

Monoclonal antibodies (mAbs) to CD68 (KP1) and rabbit polyclonal antibody to the von Willebrand factor (vWF) were purchased from DAKO (Glostrup, Denmark); rabbit polyclonal antibody to CypA was from BIOMOL International (Plymouth Meeting, PA, USA); mAb for CD147 (clone MEM-M6/1) was from Abcam (Cambridge, MA, USA); rabbit polyclonal antibody to MMP-9 was from Chemicon (Temecula, CA, USA); mAb for TFIIB (clone 24/TFIIB) was from BD-Pharmingen (San Jose, CA, USA); rabbit polyclonal antibody to IκB, mAb to phospho-IκB (Ser32/36) (5A5), PD08059, U0126, and polyclonal antibodies for ERK, phosphospho-ERK, p38, phospho-p38, AKT, and phospho-AKT (Ser473) originated from Cell Signaling (Danvers, MA, USA); SB203580, LY294002, JNK inhibitor I (JNK-I1), a cell-permeable fusion protein containing 20 AA of the JNK-binding domain of islet-brain and HIV-TAT_48-57_ (25), and its negative control containing only HIV-TAT were obtained from Calbiochem International Inc. (La Jolla, CA, USA); TPCK, ethyl pyruvate, and sulfasalazine were purchased from Sigma (St. Louis, MO, USA); and mAb for NF-κB p65 subunit (F-6) and rabbit polyclonal antibodies for p50 and goat polyclonal antibody for actin were purchased from Santa Cruz (Santa Cruz, CA, USA). Human monocytic leukemia cell line THP-1 (26) was obtained from the American Type Culture Collection (Rockville, MD, USA).

### Histological analysis

Carotid endoarterectomy specimens, generously provided by Dr. Jeong-Euy Park, Sungkyunkwan University, School of Medicine, were obtained from patients, aged between 63 to 81, who had undergone surgery at the Samsung Seoul Hospital. The current study was approved by the internal review board. Atherosclerotic plaque specimens were washed with saline and embedded to produce frozen sections. For the immunohistochemical analysis, standard 5-µm sections were stained using an LSAB kit (DAKO, Glostrup, Denmark) according to the manual provided by the manufacturer. The sections were then counterstained with Hematoxylin which stains the nucleus in blue. Finally, the slides were mounted in a 1:1 mixture of Xylene and Malinol (Muto Pure Chemicals, Tokyo, Japan).

### Cell stimulation, Western blot analysis and gelatin zymogram

For the activation utilizing immobilized mAbs, 100µl/well of PBS containing 1 or 10µg/ml of antibody was incubated overnight on a 96-well plate. The wells were washed twice with PBS, after which THP-1 cells (1×10^5^/well) in 100µl of RPMI1640 medium supplemented with 0.1% serum were added. Cell lysates were prepared at appropriate times after activation in 100µl of triple-detergent lysis buffer. For the detection of nuclear proteins, cell lysate were prepared in 200µl of NP-40 lysis buffer (0.1% NP-40, 25 mM KCl, 5 mM MgCl_2_, 10 mM Tris (pH 8.0), 1 mM PMSF, 1 mM Na_3_VO_4_, and 1 mM NaF). Cell debris containing nucleus was collected and nuclear extracts were isolated in 100µl of high salt lysis buffer (0.1% NP-40, 500 mM NaCl, 5 mM EDTA (pH 8.0), 10 mM Tris (pH 8.0), 1 mM PMSF, 1 mM Na_3_VO_4_, and 1 mM NaF). For the analysis of MMP-9, culture supernatants were concentrated 10-fold using a speedvac. Western blot analysis was performed as described previously (27). For the detection of MMP-9 using gelatin zymogram, culture supernatants were collected 24 hours after activation. The MMP-9 activity in the culture supernatant was determined by substrate gel electrophoresis as described previously (28).

### RT-PCR

Five micrograms of total RNAs isolated from cells were treated with RNase free DNase (BD-Pharmingen), and then used to generate first-strand cDNAs using a RevertAid™ first strand cDNA synthesis kit with 500 ng oligo (dT)_12-18_ primers. PCR primers were designed with ABI PRISM Primer Express 2.0 (Applied Biosystems, Foster City, CA, USA) and made by Geno Tech Corp (Daejeon, Korea). Primer sequences are 5'GGCCAGAAAACGGAGTTCAA 3' (forward) and 5' GCGCTTCTCGTAGATGAAGA 3' (reverse) for CD147, 5' ATCACTGCCACCCAGAAGAC 3' (forward) and 5' TGAGCTTGA CAAAGTGGTCG 3'(reverse) for GAPDH. After the PCR reaction, the PCR products were run on 2% agarose gel to confirm the size and purity of the PCR products.

### Immunofluorescence assay

The detection of intracellular localization of NF-κB p50 subunit was performed as described previously (20). Briefly, THP-1 cells were stimulated and fixed with 10µl of 4% formaldehyde in PBS at appropriate time after stimulation. The fixed cells were then permeabilized with 1% Triton X-100 in PBS for 10 minutes at room temperature and the permeabilized cells were then stained with 0.5µg/ml Hoechst staining solution (Sigma, St. Louis, MO, USA) for 30 minutes at 37℃ and then washed. The cells were then sequentially treated with 10µg/ml anti-p50 polyclonal antibody for 45 minutes at 37℃ and with a 1:50 dilution of Alexa Fluor 594-labeled goat anti-rabbit antibody (Invitrogen, Carlsbad, CA, USA) for 45 minutes at 37℃ in a humid chamber. Finally, the cells were dried at room temperature and mounted in a 1:1 mixture of xylene and malinol.

### Flow cytometry analysis

For the flow cytometric analysis of cell surface antigens, cells (5×10^5^) were sequentially incubated with 0.3µg of anti-CD147 mAb and FITC-labeled goat anti-mouse IgG in 30µl of FACS solution (a PBS containing 0.5% BSA and 0.1% Sodium Azide) for 20 minutes on ice. For background fluorescence, the cells were stained with an isotype-matching control antibody. The fluorescence profiles of 2×10^4^ cells were collected and analyzed using FACS-calibur (Becton-Dickinson, Mountain View, CA, USA).

## RESULTS

**Figure 1:**
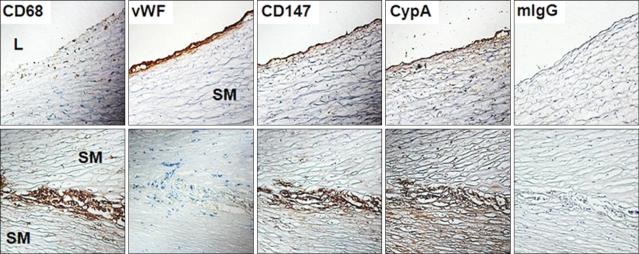
Endothelial cells and macrophages express CD147 and CypA in atherosclerotic plaques. Human carotid atherosclerotic plaques were sequentially sectioned and stained against CD68 (a macrophage marker), vWF (an endothelial cell marker), CD147, CypA. Mouse IgG (mIgG) was used for the staining as a negative control. Upper panel shows the plaques area facing the vessel lumen (L). Lower panel shows the shoulder area of a plaque which have macrophages and smooth muscle cells (SM). Note the low level staining of CypA in areas rich in SMCs.

In order to analyze the role of CD147 and its ligand (CypA) in atherogenic processes, human carotid atherosclerotic plaques were analyzed using immunohistochemical analysis (Fig. 1). The innermost layer of atherosclerotic plaque facing the lumen was lined with endothelial cells which are specifically stained with mAb against the von Willebrand factor (vWF). vWF is a multimeric glycoprotein essential for thrombus formation and the plasma level of it has been shown to be elevated in patients with atherosclerosis (29,30). Since the expression of vWF is restricted to platelets and endothelial cells, its presence has been used as a endothelial cell marker in a number of studies employing immunohistochemistry (31,32). Macrophages, stained with anti-CD68 mAb, were found in large numbers at the shoulder region of plaques in between thick layers of SMCs (Fig. 1, lower panel). CD147 expression was detected in the innermost layers facing the lumen, which corresponds to endothelial cells (Fig. 1, upper panel), and in the area corresponding to macrophage-rich regions in the shoulder region (Fig. 1, lower panel). The expression of CD147 in SMCs was not detected. Interestingly, the expression pattern of CypA, the ligand for CD147, was similar to that of CD147: both endothelial cell- and macrophage-rich areas.

**Figure 2:**
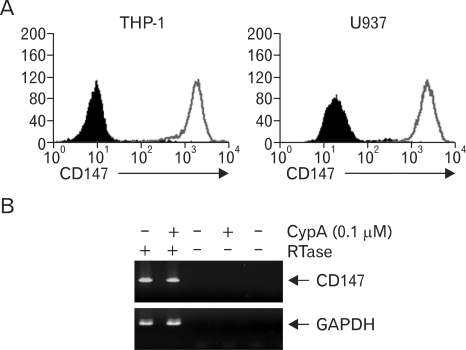
Human monocyte/macrophage cell lines express high levels of CD147. (A) THP-1 and U937 cells were stained with anti-CD147 mAb (empty area) or isotype-matching mouse IgG (filled area). (B) THP-1 cells were stimulated with or without indicated amounts of CypA for 20 hr. Total cellular RNAs were collected and the expression levels of CD147 or GAPDH mRNA were measured using RT-PCR analysis.

**Figure 3:**
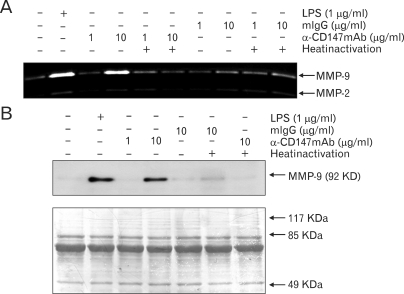
The stimulation of CD147 induced the expression of MMP-9 in THP-1 cells. (A) THP-1 cells were stimulated with anti-CD147 mAb or isotype-matching mouse IgG (mIgG) that had been immobilized at indicated concentrations. LPS was used as a positive control. Some of the cells were also stimulated with antibodies that had been heat inactivated at 95℃ for 2 hr. Culture supernatants were collected 24 hr after activation and subjected to gelatin zymogram. (B) THP-1 cells were stimulated as in (A) and culture supernatants were concentrated (×10) and subjected to Western blot analysis using MMP-9 specific antibody. As loading control, the picture of the membrane used for Western blot analysis that had been stained with Coomassie Brilliant Blue is shown at the lower panel.

Since macrophages in atherosclerotic plaques express CD147, monocyte/macrophage cell lines were used to test whether they express CD147. As shown in Fig. 2A, both THP-1 and U937 cells expressed high levels of CD147. Stimulation of THP-1 cells with CypA did not affect the expression levels of CD147, probably because the basal expression level of CD147 was already high (data not shown). The expression of CD147 in THP-1 was also confirmed using RT-PCR (Fig. 2B). THP-1 cells were then used to study the signaling pathway initiated from CD147. Since stimulation of THP-1 cells with CypA induced the expression of MMP-9 (20), CD147 on the surface of THP-1 cells were stimulated with anti-CD147 mAb and the cellular responses were analyzed. Anti-CD147 mAb was used instead of CypA, to exclude the possibility that CypA may stimulate other yet unknown cellular receptors. Stimulation of the cells with immobilized anti-CD147 mAb induced the secretion of MMP-9 (Fig. 3A). The expression levels of MMP-2, which is known to be unaffected by cellular activation status, are shown as the internal control. Isotype-matching mouse IgG failed to induce the expression of MMP-9 indicating that the induction of MMP-9 requires specific interaction between CD147 and the antibody. Furthermore, heat inactivation of anti-CD147 mAb abolished the effect indicating that the activation was not induced by endotoxins that are heat resistant. The induction of MMP-9 expression was also confirmed in the protein level using Western blot analysis (Fig. 3B).

**Figure 4:**
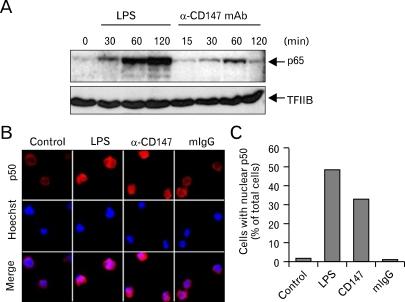
The stimulation of CD147 induces nuclear translocation of NF-κB p65 and p50 subunits. (A) THP-1 cells were stimulated with 1µg/ml of LPS or anti-CD147 mAb that had been immobilized at a concentration of 10µg/ml. Nuclear extracts were collected after indicated times and the Western blot analysis was performed using p65- or TFIIB-specific antibodies. TFIIB was used as a loading control for nuclear extracts. (B, C) THP-1 cells were stimulated with LPS, anti-CD147 mAb, or isotype-matching mouse IgG (mIgG) for 2 hr and analyzed with immunofluorescence analysis using an antibody specific for NF-κB p50 subunit. (B) shows representative pictures of cells in each samples and (C) shows the percentage of cells that had NF-κB p50 subunit at their nucleus.

The expression of MMP-9 in macrophage requires the activation of NF-κB in macrophages. NF-κB, a heterodimer of p65 and p50, stays in cytoplasm in its inactive status in association with IκB. When the activation signal(s) is transmitted, IκB become phosphorylated and, as a result, degraded by proteasome. The free NF-κB heterodimer then translocates into the nucleus. In order to analyze the requirement of NF-κB activation in the CD147-induced expression of MMP-9, immunohistochemistry and Western blot analysis was performed using p65 or p50 specific antibodies. As shown in Fig. 4A, the level of nuclear p65 was increased 30 to 60 min after activation with anti-CD147 mAb. In accordance with this data, nuclear translocation of NF-κB p50 subunit was also detected in cells stimulated with anti-CD147 mAb (Fig. 4B and 4C).

**Figure 5:**
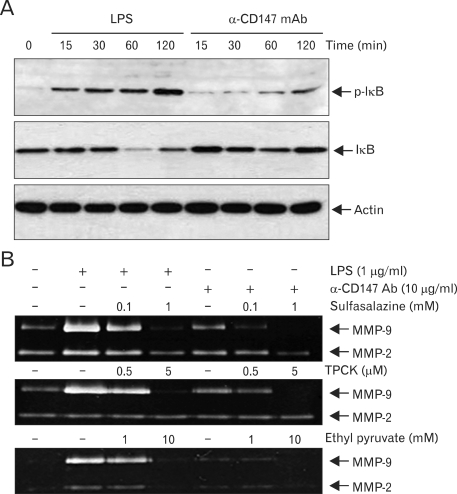
CD147-induced expression of MMP-9 requires activation of NF-κB. (A) THP-1 cells were stimulated with 1µg/ml of LPS or immobilized anti-CD147 mAb (10µg/ml). Cell lysates were collected at indicated times and subjected to Western blot analysis using antibodies specific for phospho-IκB, IκB, and actin. (B) THP-1 cells were stimulated with indicated amounts of LPS or immobilized anti-CD147 mAb in the presence of indicated amounts of NF-κB inhibitors (sulfasalazine, TPCK, or ethyl pyruvate). Culture supernatants were collected 24 hr after activation and subjected to gelatin zymogram.

The activation of NF-κB requires the phosphorylation and degradation IκB in advance. When IκB levels were analyzed after the stimulation of CD147 (Fig. 5A), phosphorylation of IκB was observed as early as 15 min after stimulation, which continued up to two hours. Accordingly, degradation of IκB was observed 30 and 60 min after stimulation. IκB level started to increase two hours after stimulation due to accumulation of newly synthesized IκB. Cells stimulated with LPS was used as a positive control. The requirement of NF-κB in CD147-induced MMP-9 secretion was also confirmed using NF-κB-specific inhibitors such as ethyl pyruvate, sulpasalazine, and *N*-tosyl-L-phenylalanine chloromethyl ketone (TPCK). These inhibitors blocked CD147-induced expression of MMP-9 in a dose-dependent manner (Fig. 5B). These data are in agreement with previous data showing the requirement of NF-κB activation for the expression of MMP-9 in CypA-treated THP-1 cells (20).

**Figure 6:**
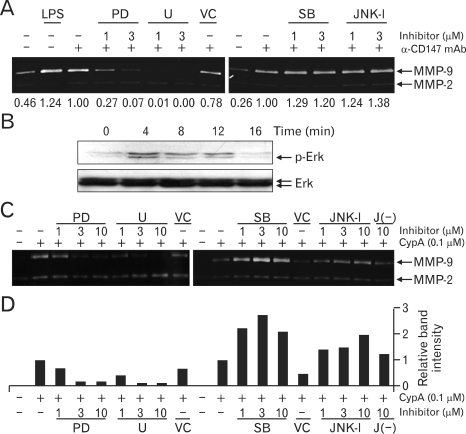
The activation of ERK is involved in the CD147-induced expression of MMP-9 and suppression of p38 and JNK activity augments the MMP-9 expression. (A) THP-1 cells were stimulated with 1µg/ml LPS or immobilized anti-CD147 mAb (10µg/ml) in the presence or absence or indicated amounts of PD98059 (PD), U0126 (U), SB203580 (SB), JNK inhibitor (JNK-I), or 0.2% DMSO as a vehicle control (VC). Culture supernatants were collected 24 hr after activation and subjected to gelatin zymogram. Numbers below each lane represent the band intensity which was normalized with band intensity of sample treated with only anti-CD147 in each gel. (B) THP-1 cells were stimulated with immobilized anti-CD147 mAb (10µg/ml). Cell lysates were collected at indicated times and the levels of phospho-ERK or ERK were analyzed using Western blot analysis. (C) THP-1 cells were stimulated with 0.1µM of CypA in the presence or absence of indicated amounts of PD98059 (PD), U0126 (U), SB203580 (SB), JNK inhibitor (JNK-I), negative control for JNK-I [J(-)], or 0.2% DMSO as a vehicle control (VC). Culture supernatants were collected 24 hr after activation and subjected to gelatin zymogram. (D) The bar graph shows the MMP-9 band intensities of each lane in panel (C) that was normalized with band intensity of sample treated with only CypA in each gel.

CypA has been reported to induce the activation of ERK1/2 in various cell types such as cancer cells, neurons, and leukocytes (21,33-35). In order to verify the involvement of MAPKs for the expression of MMP-9 in cells stimulated with CD147, the assay was performed in the presence of MAPK inhibitors. Inhibitors of ERK MAPK (U0126 and PD98059) blocked the secretion of MMP-9 in a dose-dependent manner (Fig. 6A, note the numbers below each lane). The involvement of ERK in CD147-mediated signaling was further confirmed by detecting the phosphorylation of ERK using Western blot analysis in cells stimulated with anti-CD147 mAb (Fig. 6B). Interestingly, treatment with inhibitors of p38 and JNK MAPK slightly induced MMP-9 expression (Fig. 6A, note the members below each lane which represent the relative band intensity). The involvement of MAPKs was further confirmed in THP-1 cells stimulated with CypA. As shown in Fig. 6C, ERK inhibitors suppressed MMP-9 expression in a dose dependent manner, while the inhibitors of p38 and JNK enhanced the secretion of MMP-9 (Fig. 6C and 6D). The enhancement of ERK signaling by the suppression of p38 and/or JNK has been previously reported: the presence of p38 inhibitor caused an increase in basal phosphorylation level of ERK, which resulted in the enhanced ERK-mediated signaling and cellular responses in THP-1 cells (36,37). Similar enhancement of ERK phosphorylation in the presence of p38 and JNK inhibitors is likely the cause of the observed phenomenon. The molecular mechanism underlying this suppression of ERK activity by p38 and JNK is not known.

## DISCUSSION

The immunohistochemical analysis of human atherosclerotic plaques provided the first demonstration showing the co-localization of CD147 and CypA in atherosclerotic plaques. The endothelial expression of CD147 has been previously demonstrated in cultured cells (38) and in blood brain barrier (39) and the result in figure 1 provides the first demonstration of endothelial expression of CD147 in a pathological tissue sample. Although the expression of CD147 was detected in cultured SMCs (38), it was not expressed in SMCs of atherosclerotic plaques that had been tested in this study. The co-localization of CypA and vWF indicates that endothelial cells may express CypA. Endothelial cells in atherosclerotic plaques are in highly activated status and express activation markers such as adhesion molecule ICAM-1, proinflammatory cytokines, and chemokines (40,41). Since CypA was shown to be expressed via activation in inflammatory cells (18), it is likely that endothelial cells in atherosclerotic plaque expressed high levels of CypA through a similar activation process. In case of macrophages, a number of studies already demonstrated the expression of CD147 in these cells (4,11,24) and the activation of them has been shown to induce the secretion of CypA (16). CypA is a well-known stimulator of both macrophages (20,21) and endothelial cells (17,38). These previous studies, considered together with this current data, suggest that CypA expressed by endothelial cells and macrophages can stimulate itself through CD147 in an autocrine manner. Interestingly, low level staining of CypA was detected in an area rich in SMCs, although these cells do not express CD147 (Fig. 1, lower panel). The expression of CypA by SMCs is in agreement with previous observations which reported the expression of CypA in SMCs of mouse atherosclerotic plaques (17) and in SMCs that had been activated with endotoxins (18).

The activation of ERK, after the stimulation of CD147, was detected 4 min after activation and lasted only 4 more minutes. The degradation of IκB and NF-κB nuclear translocation peaks at later time points. This indicates that ERK activation may be the upstream signaling event that leads to the degradation of IκB and subsequent NF-κB activation. Several attempts, however, failed to reveal the linear relationship between ERK and IκB/NF-κB signaling pathway; these attempts included the measurement of IκB phosphorylation/degradation and NF-κB nuclear translocation in the presence of ERK inhibitors. This may indicate that the activation of ERK stimulates MMP-9 expression in a separate pathway that does not involve NF-κB. Alternatively, ERK may activate NF-κB activation through other mechanisms such as enhancement of p65 phosphorylation, which is known to accompany the NK-κB activation and nuclear translocation and responsible for the recruitment of coactivators such as p300 (42,43).

Our data indicate that CD147 and its ligand, CypA, are expressed in endothelial cells and macrophages. Furthermore, the stimulation of CD147 that are expressed on macrophages induces ERK- and NF-κB-mediated expression of MMP-9. Macrophages play an essential role in atherogenesis through differentiation into foamy macrophages, secretion of proinflammatory cytokines/chemokines and growth factors, and enhancing thrombus formation through the expression of tissue factors, etc. Furthermore, MMPs produced by macrophages are responsible for the degradation of extracellular matrix (ECM). Degradation of ECM proteins results in weakening of the integrity of the plaque and leads to a plaque rupture and subsequent events leading to blockage of blood vessels (41). The inflammatory activation of macrophage is mediated by various mediators of inflammation such as proinflammatory cytokines, chemokines, and cell-cell interaction between inflammatory cells. The autocrine interaction between CD147 and secreted CypA is expected to contribute to and enhance the expression of MMP-9 in macrophages, which can destabilize atherosclerotic plaques by degrading ECM proteins.
